# Gene characteristics predicting missense, nonsense and frameshift mutations in tumor samples

**DOI:** 10.1186/s12859-018-2455-0

**Published:** 2018-11-19

**Authors:** Ivan P. Gorlov, Claudio W. Pikielny, Hildreth R. Frost, Stephanie C. Her, Michael D. Cole, Samuel D. Strohbehn, David Wallace-Bradley, Marek Kimmel, Olga Y. Gorlova, Christopher I. Amos

**Affiliations:** 1 The Geisel School of Medicine, Department of Biomedical Data Science, Dartmouth College, HB7936, One Medical Center Dr., Dartmouth-Hitchcock Medical Center, Beirut, NH 03756 Lebanon; 2 Department of Statistics, Rice University, M.S. 138, 6100 Main Street, Houston, TX 77005 USA

**Keywords:** Catalog of somatic mutations in Cancer, COSMIC, Somatic mutations, Missense, Nonsense, Frameshift mutations, Cancer genes

## Abstract

**Background:**

Because driver mutations provide selective advantage to the mutant clone, they tend to occur at a higher frequency in tumor samples compared to selectively neutral (passenger) mutations. However, mutation frequency alone is insufficient to identify cancer genes because mutability is influenced by many gene characteristics, such as size, nucleotide composition, etc. The goal of this study was to identify gene characteristics associated with the frequency of somatic mutations in the gene in tumor samples.

**Results:**

We used data on somatic mutations detected by genome wide screens from the Catalog of Somatic Mutations in Cancer (COSMIC). Gene size, nucleotide composition, expression level of the gene, relative replication time in the cell cycle, level of evolutionary conservation and other gene characteristics (totaling 11) were used as predictors of the number of somatic mutations. We applied stepwise multiple linear regression to predict the number of mutations per gene. Because missense, nonsense, and frameshift mutations are associated with different sets of gene characteristics, they were modeled separately. Gene characteristics explain 88% of the variation in the number of missense, 40% of nonsense, and 23% of frameshift mutations. Comparisons of the observed and expected numbers of mutations identified genes with a higher than expected number of mutations– positive outliers. Many of these are known driver genes. A number of novel candidate driver genes was also identified.

**Conclusions:**

By comparing the observed and predicted number of mutations in a gene, we have identified known cancer-associated genes as well as 111 novel cancer associated genes. We also showed that adding the number of silent mutations per gene reported by genome/exome wide screens across all cancer type (COSMIC data) as a predictor substantially exceeds predicting accuracy of the most popular cancer gene predicting tool - MutsigCV.

**Electronic supplementary material:**

The online version of this article (10.1186/s12859-018-2455-0) contains supplementary material, which is available to authorized users.

## Background

Predictive differentiation between functional and neutral somatic and germline mutations was and continues to be a hot topic of bioinformatics research. A number of tools using a number of predictors including, level of evolutionary conservation, effect on protein structure, functional DNA sequences, e.g. transcription factor binding sites and other have been developed [1–7]. However, more specific topic, namely development of tools for identification of cancer-associated genes gets less attention.

In many cases cancer development is driven by somatic mutations. [8] Mutations providing a proliferative or survival advantage to the mutant clone (drivers) occur more frequently in tumor samples compared to selectively neutral (passenger) mutations. [9, 10] Known cancer-associated genes are among the most frequently mutated genes. In general, the number of somatic mutations per gene indicates the gene’s involvement in cancer development. However, a simple counting of somatic mutations can be misleading because the number of mutations per gene depends not only on the involvement of the gene in tumorigenesis but also on the gene’s intrinsic mutability that in turn depends on gene characteristics.

A number of gene characteristics have been shown to be associated with mutability. It has been shown that genes with a higher expression level tend to have a higher frequency of somatic mutations. [11, 12] Another known gene characteristic associated with mutability is relative replication time within cell cycle: later replicating genes tend to have a higher number of somatic mutations. [11, 12] Chromatin accessibility has been shown to be positively associated with the density of somatic mutations. [13] Differences in mutation rate of different nucleotide substitutions, e.g. high frequency of transitions in CpG sites [14] suggest that nucleotide composition of the gene also may be associated with mutability. Those and other gene characteristics are inter-correlated. Gene length has been shown to be correlated with selective codon usage (nucleotide composition) [15] Replication timing is correlated with gene expression level [16] We found that size of the gene positively correlates with the level of evolutionary conservation. [17] Inter-correlations between predictors call for a multivariate regression model to predict the number of somatic mutations in the gene. According to our initial analyses, missense, nonsense and frameshift may have different sets of predictors (gene characteristics) and therefore need to be modeled separately. A recent study by Martincorena et al. [18] used normalized ratio of non-synonymous to synonymous mutations to identify genes under positive or negative selection in cancer evolution. The authors noted that about half of the identified driver mutations “occur in yet-to-be-discovered cancer genes”.

Our analysis is based on the hypothesis that inter-gene variation in the number of somatic mutations has two sources: (1) the variation due to differences in gene characteristics, and (2) the variation due to the involvement of the gene in cancer development. We tried to explain the intergenic variation in the number of somatic mutations by the variation in gene characteristics. Outliers – genes for which the number of somatic mutations cannot be explained by gene characteristics are candidate cancer genes.

## Methods

### Design of the study

The goal of this study is to build statistical model for prediction of the expected number of somatic mutations in a given gene based on the gene characteristics. To build the model we used somatic mutation data generated by whole exome sequencing of tumor samples. We separately predicted missense, nonsense, and frameshift mutations. Residuals from the models were analyzed to detect outliers – genes with a higher-than-expected number of mutations. The excess of mutations unexplained by gene characteristics is due to the gene involvement in cancer development and can be used to identify cancer-associated genes.

### Mutation data

We used mutation data from the Catalog of Somatic Mutations in Cancer (COSMIC) (accessed August 17, 2017). To ensure uniform testing across all genes, only mutations detected by whole genome screens were used. All cancer types were included in the analysis. A total of 19,147 tumor samples were analyzed. Mutations reported as SNPs were excluded from the analysis. In total there were 2,233,115 missense, 163,823 nonsense, and 85,272 frameshift (FS) mutations, including those resulted from nucleotide insertions as well as nucleotide deletions.

### Gene characteristics

The following gene characteristics were used as predictors:

1. *Gene size.* We used data from the NCBI Consensus coding sequence project to estimate gene coding region sizes. [19] When multiple transcripts were reported for the same gene, the largest transcript was used. A moving average was used to illustrate the relationship between the gene size and the number of somatic mutations in it. In brief, genes were ranked based on the size from shortest to longest. The sliding window of 100 nucleotides was moved along the genes with one nucleotide step. We found that this size of the sliding window is optimal for smoothing of the relationship while keeping the effects of strong outliers like *TP53* visible. The average size and average number of mutations were computed for each position of the window. Scatterplots were used to visualize the relationship between the gene size and the number of mutations. The moving average approach was used to visualize the relationships between the number of mutations in the gene and other predictors.
2. *Number of potential sites for a given type of mutations.* The type of mutation produced by a single nucleotide substitution (SNS) depends on type of SNS (e.g. C > T) and its position in a given codon. There are three possible SNSs per each nucleotide position which makes the total number of all possible SNSs in the gene equal to 3xN, where N is the length of the coding region in nucleotides. We predicted outcomes of all possible SNSs in each gene to estimate the number of SNSs producing missense, nonsense or silent mutations in the gene – the number of potential sites in a gene for a given type of somatic mutations.
3. *Nucleotide composition.* For each gene we estimated the proportions of each of the four nucleotides in the coding region of the gene. The relationship between the percentage of each nucleotide and mutation densities were analyzed. Mutation densities were computed as the ratios of the total number of mutations to the size of the coding region of the gene in nucleotides. We used the density rather than the number of mutations per gene to account for the effect of the gene size.
4. *Percentage of CpGs.* Mutation rate is known to be higher in CpG dinucleotides [14] suggesting that genes with a higher proportion of CpG may have a higher mutation rate and as a result a higher number of somatic mutations. We used percentage of CpGs as a predictor of mutation density.
5. *Evolutionary conservation.* Some studies indicate that evolutionary conservation of the gene correlates with mutability. [20] As a measure of evolutionary conservation of the gene we used conservation index. [21] Orthologs for each gene were identified among 20 species with complete genome sequences: *Pan troglodytes, Macaca mulatta, Canis lupus familiaris, Bos taurus, Mus musculus, Rattus norvegicus, Gallus gallus, Xenopus tropicalis, Danio rerio, Drosophila melanogaster, Anopheles gambiae, Caenorhabditis elegans, Saccharomyces cerevisiae, Kluyveromyces lactis, Eremothecium gossypii, Schizosaccharomyces pombe, Magnaporthe oryzae, Neurospora crassa, Arabidopsis thaliana*, and *Oryza sativa*. Conservation index of 1 was assigned to the genes with 0 or 1 orthologs, conservation index 2 was assigned to the genes with 2 or 3 orthologs and so on.
6. *Gene expression level*. It has been shown that the expression level of the gene negatively correlates with the density of somatic mutations. [11, 12] Gene expression data for 1037 cancer cell lines were downloaded from the Cancer Cell Line Encyclopedia (CCLE). [22] For each gene we computed average expression across CCLE cell lines and used it as a predictor of the mutation density.
7. *Nucleotide diversity*. We noted bell-shaped curves describing the relationship between the percentage of nucleotides and the density of missense mutations suggesting that genes with similar percentages of all nucleotides (25% each) may tend to have a higher density of somatic mutations. To account for this effect we devised a single measure characterizing how strongly the proportions of four nucleotides deviate from being equal. We called this measure nucleotide diversity (ND). ND was defined as the probability that two nucleotides randomly selected from the gene coding sequence are different: ND = 1-(P_(A)_^2^ + P_(C)_^2^ + P_(G)_^2^ + P_(T)_^2^), where P_(A)_, P_(C)_, P_(G)_, and P_(T)_ are the percentages of each nucleotide in the gene. ND was computed for each gene and used as a predictor.
8. *SNP density*. Genes with a high propensity to mutate are also expected to have a higher density of germline polymorphisms. We used SNPs to estimate the density of germline polymorphisms in a gene. SNP density was computed as a ratio of the total number of unique SNPs in the coding region to its size in nucleotides. SNPs detected by the 1000 genomes project [23] were used in this analysis to ensure that different genes were targeted the same number of times.
9. *Density of the silent mutations*. Even though some silent mutations are known to be functional [24], most of them are neutral and therefore the density of silent mutations in the gene can be used as a quantitative measure of mutability of the gene. We computed the density of silent mutations for each gene and used it as a predictor.
10. *Relative replication time*. Late-replicating genes tend to have a higher number of mutations. [11, 12] We used the relative replication time data from Ryba et al. (2012). [25] Human genome build GRCh38 was used to match the positions of probes with positions of the genes. When several probes were mapped to the same gene, average replication time for all probes in the gene was used as a predictor. The closest probe was used when there were no probes in the gene. The relative replication time (negative for early and positive for late-replicating genes) was used as a predictor.
11. *Chromatin accessibility*. Chromatin accessibility has been shown to be associated with mutability of the region. [13] Data from the study by Sos et al. [26] were used in chromatin accessibility analysis. The study used transposon hypersensitive sites sequencing assay to assess chromatin accessibility. The mean chromatin accessibility across 10 lymphoblastic cell lines was computed for each gene and used as a predictor for density of missense, nonsense and FS mutations separately.
12. *Covariates from MutsigCV*. We also included three predictors (co-variates) used by MutsigCV: “expr”, “hic” and “reptime” [12]. “Expr” is the expression level of this gene, averaged across 91 cell lines in the Cancer Cell Line Encyclopedia. “Reptime” is replication time of this gene (measured in HeLa cells), ranging from 100 (very early) to 1000 (very late). “Hic” chromatin state of this gene (measured from HiC experiments in K562 cells) ranging from − 50 (very closed) to + 50 (very open). We used similar predictors gene expression, relative replication time and chromatin accessibility. The difference of our predictors from those used by MutsigCV was sources of the data: we used different studies to estimate the same gene characteristics. By using different sources we can assess the reliability of the predictors and their sensitivity to the source of the data.

### Statistical analysis

As a first step for statistical analysis we examined descriptive statistics for predictors and outcome and estimated pairwise correlations between predictors across 15,610 genes. We used non-parametric Spearman’s rank order correlation. We used a stepwise multiple linear regression model implemented in STATISTICA (StatSoft) to identify a best subset of predictors of the number of mutations per gene. Residual analysis was used to detect outliers – genes with a higher than expected number of missense, nonsense, or FS mutations. For each gene, residual Z-scores were computed separately for missense, nonsense and FS mutations. Residuals from the prediction models follow standard normal distribution N(0,1). Z-score is the signed value of standard deviations from mean which is zero for standard normal distribution. Positive Z-score indicates an excess and negative - a deficit of mutations in the gene compared to the expected numbers. The absolute value of Bonferroni corrected Z values based on 15,610 tests (the total number of genes used in the analysis) was further corrected as being a maximum of three Z-scores. Only genes with complete data for all predictors were used in this analysis. Under the assumption of independence of the 3 scores, the threshold used for significance was: ${\Phi}^{-1}\left(\sqrt[3]{1-\alpha /n}\right)$, where Φ^−1^(*p*) denotes the quantile function of the normal distribution, *α* = 0.05 and *n* = 15,610, which yielded a cutoff value of 4.74.

## Results

**Fig. 1:**
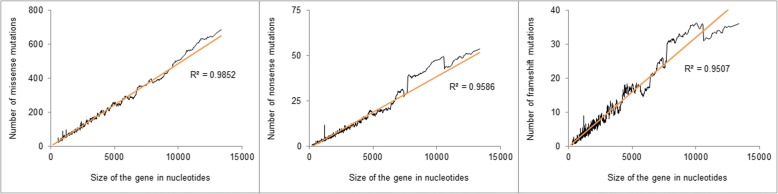
The relationship between the number of missense, nonsense, and frameshift mutations and gene size

As expected, strong positive associations between the gene size and the number of mutations were detected for all types of mutations (Fig. 1). Similar relationships were detected with the number of potential sites (Additional file 1).

**Fig. 2:**
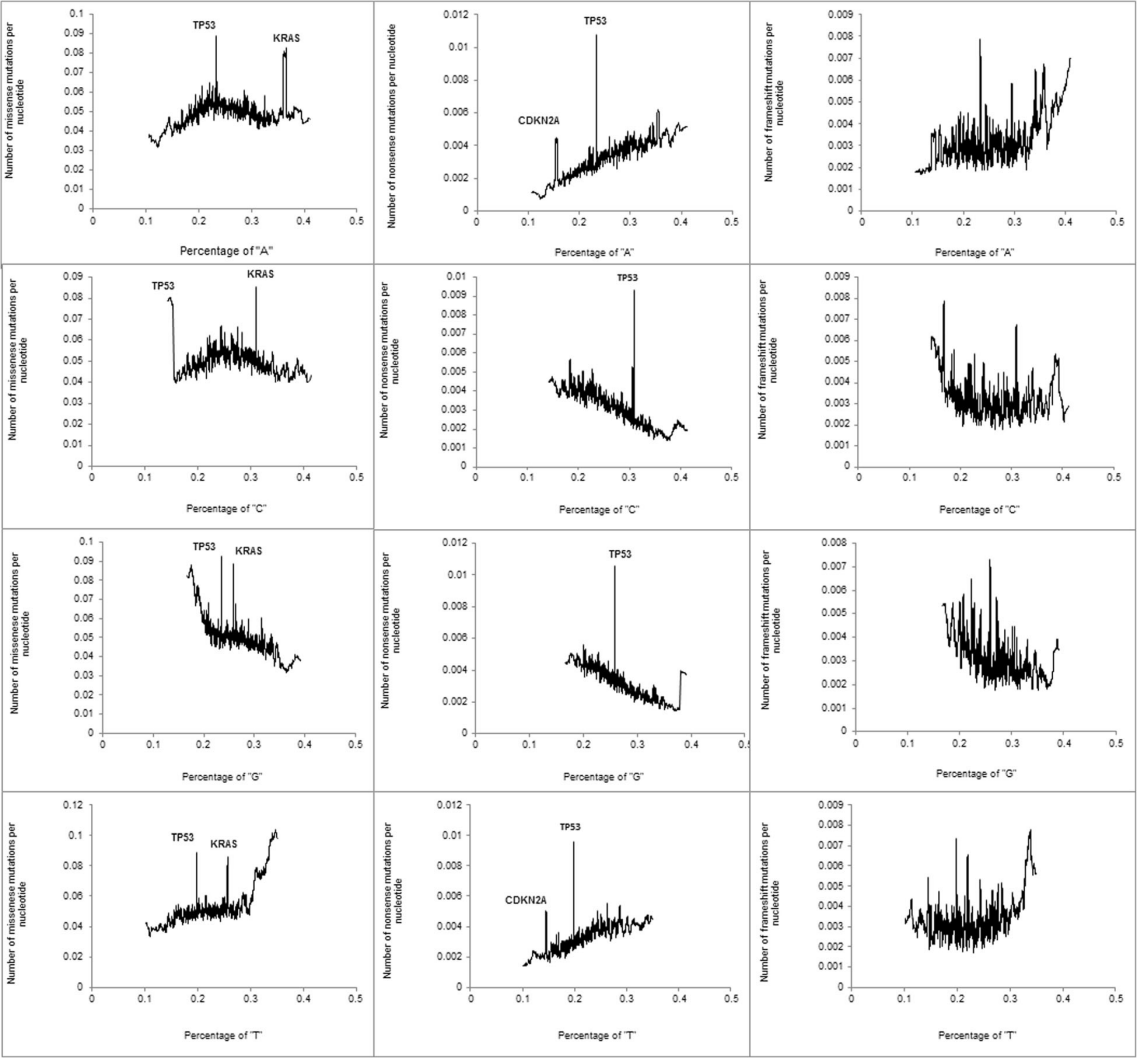
The relationship between the nucleotide composition and the density of missense (first column), nonsense (second column), and FS (third column) mutations

Figure 2 shows the relationship between the nucleotide composition and the density of missense (first column), nonsense (second column) and frameshift (third column) mutations. For nonsense mutations, there was a linear relationship between the percentage of each nucleotide and the mutation density, as expected from the nucleotide composition of stop codons (TAA, TAG, and TGA). Peaks on the curves are driven by *CDKN2A* and *TP53*. These genes have a much larger number of nonsense mutations compared to the genes with a similar nucleotide composition. For missense mutations, the peaks are driven by *TP53* and *KRAS*. A curvilinear shape describes the relationships between the percentages of “A” and “C” nucleotide percentage and density of missense mutations. The peak coincides with nucleotide densities close to 0.25.

We observed an up-going tail on the left side of the curve describing the relationship between the percentage of “G” and the density of missense mutations. A similar up-going tail was observed on the right side of the curve describing the relationship between the percentage of “T” and the density of missense mutations. Both tails are driven by olfactory receptor genes (total 368). We found that the density of missense mutations in olfactory receptors is twice that of other genes in the human genome: 107.5 ± 2.9 versus 49.4 ± 0.4 mutations per 1 kb. Densities of nonsense and FS mutations in olfactory genes are not elevated. Olfactory genes also have an unusually low percentage of “G” and a high percentage of “T”. The percentages of “A”, “C”, “G” and “T” in olfactory genes are correspondingly 22.1 ± 0.3, 26.6 ± 0.3, 20.2 ± 0.3, and 31,1 ± 0.3, while the corresponding percentages in all other genes are 24.3 ± 0.1, 26.3 ± 0.1, 27.8 ± 0.1, and 21.6 ± 0.1. The combination of an “abnormal” nucleotide composition and a higher density of missense mutations result in up-going tails for missense mutations: left for the percentage of “G” and right for the percentage of “T”. When olfactory genes were removed from the analyses, the up-going tails disappeared (Additional file 2).

For frameshift mutations, we detected a positive linear relationship between the percentage of “A” and the density of mutations and a negative relationship with the percentage of “G”. Densities of missense and nonsense mutations were negatively associated with both the percentage of CpGs and the level of evolutionary conservation (Additional files 3 and 4, respectively).

**Fig. 3:**
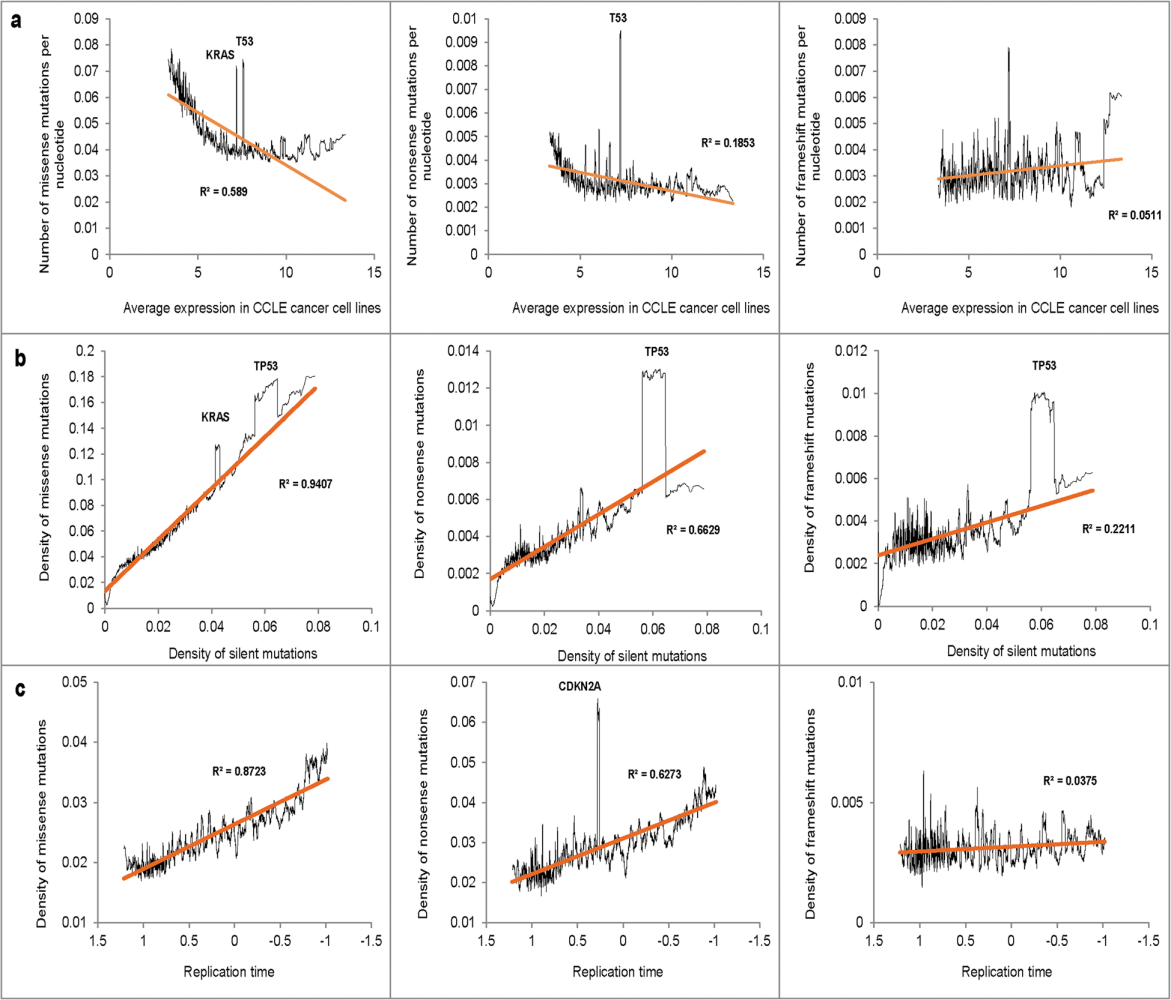
**(a)** The relationship between average expression in CCLE cancer cell lines and the mutation densities. (**b)** The relationship between the density of silent mutations and the densities of missense, nonsense and frameshift mutations. (**c)** The relationship between the relative replication time and the densities of missense, nonsense, and frameshift mutations

We observed a negative association between the average expression level in CCLE cancer cell lines and the mutation densities (Fig. 3a). Because the curves were L-shaped, we log-transformed gene expression values. The transformation improved the R^2^ derived from linear regression from 0.59 to 0.69 for missense, and from 0.18 to 0.27 for nonsense mutations. Correlation between gene expression and the density of frameshift mutations was not significant. We also noted a strong positive association between the density of silent mutations in the gene with the densities of other mutation types (Fig. 3b). Figure 3c shows the relationship between the mutation densities of missense, nonsense and FS mutations and the relative replication time. Consistent with published studies [11, 12] we observed a strong positive association between the replication time and the mutation density for missense and nonsense mutations but not for frameshift mutations.

A positive association between the nucleotide diversity (ND) and the densities of missense and nonsense mutations was noted (Additional file 5). A significant negative association between chromatin accessibility and the density of missense and nonsense mutations in the gene has been observed (Additional file 6).

### Correlations between predictors

We found that gene characteristics used in this analysis are highly correlated (Table 1). Out of 120 possible pair wise correlations, 112 pairs were statistically significant. Aside from expected correlations, e.g. correlation between the number of potential sites for mutations and gene size, we observed a number of unexpected correlations. For example, we noted that larger genes tended to have a higher percentage of “A” nucleotides. Larger genes also tended to have higher evolutionary conservation indices. Genes with a higher expression level tended to replicate earlier. Because of widespread correlations among predictors we used stepwise best subset multivariate regression.

**Table 1:** Pair-wise correlations between gene characteristics

	% “A”	% “G”	% “C”	% “T”	ND	% “CpG”	CDS	SNPD	EC	NPS	NPM	AGE	LAGE	RRT	HA	NSM
% “A”	1.00	− 0.71	− 0.88	0.47	0.35	− 0.72	0.11	− 0.22	0.09	0.27	0.12	0.19	0.18	−0.26	− 0.04	− 0.01
% “G”	−0.71	1.00	0.60	−0.78	−0.46	0.76	−0.11	0.20	−0.06	− 0.22	− 0.12	− 0.06	− 0.04	0.27	0.06	−0.03
% “C”	− 0.88	0.60	1.00	−0.69	− 0.44	0.74	− 0.06	0.22	− 0.13	− 0.22	−0.07	− 0.24	−0.23	0.25	0.04	0.06
% “T”	0.47	−0.78	−0.69	1.00	0.54	−0.71	0.04	−0.18	0.10	0.13	0.04	0.11	0.10	−0.24	−0.06	− 0.04
ND	0.35	−0.46	−0.44	0.54	1.00	−0.60	0.08	−0.09	0.16	0.12	0.09	0.10	0.09	−0.09	−0.02	0.09
% “CpG”	−0.72	0.76	0.74	−0.71	−0.60	1.00	−0.13	0.15	−0.04	− 0.24	−0.14	− 0.09	−0.08	0.19	0.04	−0.03
CDS	0.11	−0.11	−0.06	0.04	0.08	−0.13	1.00	−0.10	0.08	0.97	1.00	−0.09	−0.07	− 0.05	−0.01	0.81
SNPD	−0.22	0.20	0.22	−0.18	−0.09	0.15	−0.10	1.00	−0.17	− 0.13	−0.10	− 0.13	−0.13	0.09	0.02	0.02
EC	0.09	−0.06	−0.13	0.10	0.16	−0.04	0.08	−0.17	1.00	0.08	0.09	0.27	0.27	0.08	−0.01	0.01
NPS	0.27	−0.22	−0.22	0.13	0.12	−0.24	0.97	−0.13	0.08	1.00	0.97	−0.06	−0.04	− 0.09	− 0.02	0.74
NPM	0.12	−0.12	−0.07	0.04	0.09	−0.14	1.00	−0.10	0.09	0.97	1.00	−0.09	−0.07	− 0.05	− 0.01	0.81
AGE	0.19	−0.06	−0.24	0.11	0.10	−0.09	−0.09	− 0.13	0.27	− 0.06	−0.09	1.00	0.98	0.19	0.07	−0.26
LAGE	0.18	−0.04	−0.23	0.10	0.09	−0.08	−0.07	− 0.13	0.27	− 0.04	−0.07	0.98	1.00	0.20	0.08	−0.26
RRT	−0.26	0.27	0.25	−0.24	−0.09	0.19	−0.05	0.09	0.08	−0.09	− 0.05	0.19	0.20	1.00	0.18	−0.17
HA	−0.04	0.06	0.04	−0.06	−0.02	0.04	−0.01	0.02	−0.01	− 0.02	− 0.01	0.07	0.08	0.18	1.00	−0.05
NSM	−0.01	−0.03	0.06	−0.04	0.09	−0.03	0.81	0.02	0.01	0.74	0.81	−0.26	−0.26	− 0.17	−0.05	1.00

ND- Nucleotide diversity, CDS CDS size, SNPD - SNP density, EC - Evolutionary conservation, NPS - N potential stops, NPM - N potential missense, AGE - Average gene expression, LAGE - LOG of average gene expression, RRT - Relative replication time, HA - Chromatin accessibility, NSM - N of silent mutations

### Univariate analyses

Below we present the results of univariate regression with the number of mutations in the gene as the outcome and gene characteristics as predictors.

#### Missense mutations

In the univariate analysis, the most significant predictor of the number of missense mutations was the number of silent mutations in the gene (Table 2). Gene size and the number of potential missense mutation sites were the next most significant predictors with similar levels of significance. Relative replication time from MutsigCV (“reptime”) and our analogous predictor (relative replication time) show similar levels of significance. Our predictor “Gene expression in CCLE cancer cell lines” was more significant compared to the analogous predictor from MutsigCV – “expr”. For chromatin accessibility, MutsigCV predictor “hic” was more significant compared to our predictor “Chromatin accessibility”.

**Table 2:** Gene characteristics associated with the number of missense mutations per gene in univariate regression models

Predictor	T-test	*P*-value	Beta (ß)
Number of silent mutations in the gene	289.9	2.5 × 10^− 1409^	0.92
Number of potential missense mutation sites	167.5	5.8 × 10^− 805^	0.80
Gene size in nucleotides	167.1	1.6 × 10^− 803^	0.80
“reptime” from MutsigCV	28.6	1.4 × 10^− 126^	0.23
Relative replication time	−27.5	1.0 × 10^− 124^	0.21
Gene expression in CCLE cancer cell lines*	−26.5	9.6 × 10^− 118^	− 0.21
“hic” from MutsigCV	−24.5	6.6 × 10^− 113^	− 0.20
“expr” from MutsigCV	−16.8	1.5 × 10^− 54^	− 0.14
Percentage of “CpG”	− 16.3	1.7 × 10^− 53^	−0.02
Percentage of “G”	− 15.9	3.1 × 10^− 51^	−0.13
Nucleotide diversity	14.7	5.5 × 10^− 45^	0.12
Percentage of “A”	11.2	5.7 × 10^− 28^	0.10
Chromatin accessibility	−7.7	3.1 × 10^− 51^	−0.06
Percentage of “C”	−7.1	5.5 × 10^− 45^	− 0.06
Percentage of “T”	7.0	5.7 × 10^− 28^	0.06
Density of SNPs (1 K Genomes Project)	−3.7	1.6 × 10^− 14^	− 0.03
Evolutionary conservation	1.5	1.1 × 10^− 12^	0.01

#### Nonsense mutations

Table 3 shows results of univariate analysis for nonsense mutations. The number of potential sites for nonsense mutations was the most significant predictor, followed by the gene size and number of silent mutations. Compared to missense mutations nucleotide composition seem to be more important for the prediction of nonsense mutations. This is likely due to the fact that a subset of codons capable to produce nonsense mutations tends to be A-rich and G-poor.

**Table 3:** Gene characteristics associated with the number of nonsense mutations in the univariate linear regression model

Predictor	T-test	*P*-value		Beta (ß)
Number of potential nonsense mutation sites	91.3	3.1 × 10^− 427^		0.59
Gene size in nucleotides	84.7	7.8 × 10^− 395^		0.56
Number of silent mutations in the gene	80.0	1.6 × 10^−371^		0.54
Percentage of “A”	24.2	1.4 × 10^−102^		0.19
Percentage of “G”	−22.6	1.0 × 10^−91^		−0.18
Percentage of “CpG”	− 21.9	1.2 × 10^−87^		0.01
“reptime” from MutsigCV	20.1	2.6 × 10^−76^		0.17
Percentage of “C”	−19.6	1.0 × 10^− 72^		− 0.15
Relative replication time	− 19.4	1.7 × 10^− 71^		− 0.15
“hic” from MutsigCV	− 17.1	4.5 × 10^−58^		− 0.14
“expr” from MutsigCV	−16.0	1.1 × 10^− 51^		− 0.13
Percentage of “T”	14.4	2.7 × 10^− 43^		0.11
Nucleotide diversity	12.6	2.0 × 10^− 34^		0.10
Gene expression in CCLE cancer cell lines*	−11.2	1.1 × 10^− 27^		− 0.09
Density of SNPs (1 K Genomes Project)	−8.8	3.5 × 10^−18^		−0.07
Evolutionary conservation	5.0	3.7 × 10^−7^		0.04
Chromatin accessibility	−4.9	6.7 × 10^− 7^		−0.04

#### Frameshift mutations

Table 4 shows the results of univariate analyses for FS mutations. The gene size was the most significant predictor followed by the number of silent mutations. The nucleotide composition was also significant with C + G rich genes having lower number of FS mutations. The level of evolutionary conservation was positively associated with the number of FS mutations in the gene.

**Table 4:** Gene characteristics associated with the number of FS mutations per gene in univariate linear regression model

Predictor	T-test	*P*-value	Beta (ß)
Gene size in nucleotides	65.6	1.7 × 10^− 354^	0.46
Number of silent mutations in the gene	52.3	1.2 × 10^− 288^	0.39
Percentage of “A”	14.6	2.8 × 10^− 44^	0.12
Percentage of “G”	−14.1	1.8 × 10^− 41^	− 0.11
Percentage of “CpG”	−12.7	1.3 × 10^−34^	− 0.10
Percentage of “C”	− 7.9	2.5 × 10^− 15^	− 0.06
Evolutionary conservation	6.4	1.6 × 10^− 10^	0.34
“reptime” from MutsigCV	5.9	1.9 × 10^− 9^	0.05
Density of SNPs (1 K Genomes Project)	−5.7	7.6 × 10^− 9^	− 0.05
“expr” from MutsigCV	−4.9	5.9 × 10^− 7^	− 0.04
Relative replication time	− 4.8	9.4 × 10^− 7^	− 0.04
Percentage of “T”	4.2	1.6 × 10^−5^	0.03
“hic” from MutsigCV	− 3.4	3.4 × 10^− 4^	− 0.03
Gene expression in CCLE ancer cell lines*	−1.7	4.7 × 10^− 2^	− 0.01
Chromatin accessibility	−1.4	8.4 × 10^− 2^	− 0.01
Nucleotide diversity	0.2	4.2 × 10^− 1^	0.00

#### Prediction of the number of missense, nonsense and frameshift mutations together

Table 5 shows predictors for missense, nonsense and frameshift mutations analyzed together. The results of this analysis are similar to the results of the analysis of missense mutations.

**Table 5:** Gene characteristics associated with the number of missense, nonsense and frameshift mutations analyzed together in univariate linear regression model

Predictor	T-test	*P*-value	Beta (ß)
Number of silent mutations in the gene	265.2	3.6x^− 1328^	0.90
Gene size in nucleotides	172.5	2.7x^− 856^	0.81
“reptime” from MutsigCV	28.0	2.1x^− 128^	0.23
Relative replication time	−26.8	6.5x^− 120^	− 0.21
Gene expression in CCLE cancer cell lines*	−24.8	1.2x^− 106^	− 0.19
“hic” from MutsigCV	−23.8	8.0x^− 100^	− 0.19
Percentage of “CpG”	−17.9	1.8x^− 62^	− 0.14
Percentage of “G”	− 17.7	1.8x^− 61^	− 0.14
“expr” from MutsigCV	−17.0	3.1x^−57^	− 0.14
Percentage of “A”	15.3	7.3x^− 48^	0.12
Nucleotide diversity	14.4	4.9x^− 43^	0.11
Percentage of “C”	−9.0	5.6x^−19^	− 0.07
Percentage of “T”	8.0	1.6x^− 15^	0.06
Chromatin accessibility	− 7.5	1.0x^− 13^	− 0.06
Density of SNPs (1 K Genomes Project)	−4.7	1.6x^− 6^	−0.04
Evolutionary conservation	2.4	8.4x^−3^	0.02

### Predictors for multivariate analysis

We selected predictors for multivariable analysis based on their significance in univariate analyses and the linearity of the association with the outcome. Table 6 shows the gene characteristics selected for each type of mutations. In all multivariate analyses we also included three covariates from MutsigCV (not shown in Table 6). Olfactory genes were excluded because of their distinctive nucleotide composition and high density of missense mutations. *TP53, CDKA2*, and *KRAS* were also excluded from the analyses because they were obvious outliers in univariate analyses.

**Table 6:** Gene characteristics selected for the model building for the missense, nonsense, and frameshift mutations

Predictor	Used for		
	Missense	Nonsense	Frameshift
Density of SNPS (1 K Genomes Project)	yes	no	yes
Evolutionary conservation	yes	yes	no
Gene expression in CCLE cancer cell lines^a^	yes	yes	no
Gene size in nucleotides	yes	yes	yes
Nucleotide diversity	yes	yes	yes
Number of potential substitution sites	yes	yes	yes
Number of silent mutations in the gene	yes	yes	yes
Percentage of “A”	no	yes	yes
Percentage of “C”	yes	yes	no
Percentage of “G”	yes	yes	yes
Percentage of “T”	yes	yes	no
Percentage of “CpGs”	yes	yes	yes
Replication time	yes	yes	no
Chromatin accessibility	yes	yes	no

^a^Average gene expression across 1037 cancer cell lines from the Cancer Cell Line Encyclopedia (CCLE)

### Multivariate analysis

#### Prediction of missense mutations

Table 7 shows missense mutations predictors that remained significant in the stepwise best subset linear regression. The most significant predictor was the number of silent mutations in the gene. Nucleotide diversity and the percentages of “C” and “G” nucleotides were also significant. The R^2^ for the whole model was 0.88. Additional file 7 shows the relationship between the predicted and the observed numbers of missense mutations.

**Table 7:** Gene characteristics significant in stepwise best subset multiple linear regression model for the prediction of the number of missense mutations

Predictor	T-test	*P*-value	Beta (ß)
Number of silent mutations in the gene	136.47	1.8 × 10^− 376^	0.76
Replication time	−7.74	1.0 × 10^−14^	−0.03
Percentage of “C”	− 7.64	2.4 × 10^− 14^	− 0.06
Nucleotide diversity	−6.65	3.1 × 10^−11^	− 0.03
Percentage of “G”	− 6.14	8.2 × 10^−10^	− 0.03
“reptime” from MutsigCV	5.24	1.7 × 10^− 7^	0.02
Evolutionary conservation	−4.57	4.9 × 10^− 6^	− 0.01
Percentage of “CpGs”	− 3.62	3.0 × 10^− 4^	− 0.02
“expr” from MutsigCV	2.79	5.3 × 10^− 3^	0.01
Number of potential sites for missense mutations	2.57	1.0 × 10^−2^	0.42

#### Prediction of nonsense mutations

Table 8 shows gene characteristics that remained significant in the multiple linear regression model for nonsense mutations. The most significant predictor was the number of potential sites for nonsense mutations. The other significant predictors included number of the detected silent mutations and the gene size. The model R^2^ was 0.40. Additional file 8 shows the relationship between the predicted and the observed numbers of nonsense mutations.

**Table 8:** Gene characteristics significant in stepwise best subset multiple linear regression model for nonsense mutations

Predictor	T-test	*P*-value	Beta (ß)
Number of potential sites for nonsense mutations	27.77	3.42 × 10^−132^	0.782
Number of silent mutations in the gene	26.03	1.66 × 10^− 129^	0.301
Gene size in nucleotides	−16.22	5.10 × 10^− 49^	− 0.498
Percentage of “G”	− 3.07	4.60 × 10^− 4^	− 0.028
Replication time	−2.35	7.10 × 10^− 3^	− 0.021
Evolutionary conservation	2.23	2.26 × 10^− 2^	0.016

#### Prediction of frameshift mutations

Table 9 shows predictors that remained significant in the multiple linear regression model for FS mutations. Gene size was the most significant predictor followed by the nucleotide diversity (negative association) and the percentages of “A” and “C” nucleotides that were positively associated with the number of FS mutations in the gene. The R^2^ of the model for FS mutations was 0.23. Additional file 9 shows the relationship between the predicted and the observed numbers of FS mutations.

**Table 9:** Gene characteristics significant in stepwise best subset multiple linear regression model for frameshift mutations

Predictor	T-test	*P*-value	Beta (ß)
Gene size in nucleotides	50.93	3.34 × 10^− 218^	0.38
Nucleotide diversity	−10.69	5.56 × 10^− 26^	− 0.1
Number of silent mutations in the gene	6.86	5.65 × 10^− 09^	0.1
Percentage of “C”	5.26	3.84 × 10^−7^	0.09
Percentage of “A”	5.16	6.52 × 10^− 7^	0.09
Gene expression in CCLE cancer cell lines	4.21	5.64 × 10^−5^	0.03
Percentage of “CpGs”	− 4.1	8.84 × 10^− 5^	− 0.06
Percentage of “G”	− 3.62	5.52 × 10^− 4^	− 0.04
“hic” from MutsigCV	2.25	3.14 × 10^− 2^	0.01
Evolutionary conservation	2.09	4.42 × 10^− 2^	0.01

#### Prediction of the number of missense, nonsense and frameshift mutations together

Table 10 shows predictors for missense, nonsense and frameshift mutations analyzed together. The gene size was the most significant predictor, followed by the nucleotide diversity (negative association) and the percentage of “A” and “C” nucleotides (positive associations). The R^2^ of the model for all mutations was 86%.

**Table 10:** Gene characteristics significant in stepwise best subset multiple linear regression model for missense, nonsense, and frameshift mutations analyzed together

Predictor	T-test	*P*-value	Beta (ß)
Number of silent mutations in the gene	121.8	1.6x^− 789^	0.72
Gene size in nucleotides	41.6	1.4x^− 220^	0.23
Nucleotide diversity	−8.5	3.3x^−17^	−0.04
Relative replication time	−7.0	1.7x^−12^	−0.03
Percentage of “G”	− 7.0	2.6x^− 12^	− 0.04
Percentage of “C”	− 7.0	3.1x^− 12^	− 0.05
“reptime” from MutsigCV	5.4	4.1x^− 8^	0.03
Percentage of “CpG”	−3.7	1.0x_− 4_	− 0.02
Evolutionary conservation	− 3.3	4.7x^− 4^	− 0.01
Percentage of “A”	2.6	4.8x^− 3^	0.02
“expr” from MutsigCV	2.5	6.4x^− 3^	0.01

### Mutation type specific models

We tested how well the pan-mutation model works for predicting missense, nonsense and FS mutations separately. We compared them with mutation type specific models by the prediction accuracy. R^2^s were used to evaluate how well the model accounts for gene characteristics. R^2^s were computed by comparison of the observed and predicted number of mutations in the genes.

The pan-mutation model predicts missense mutations almost as well as the missense-specific model described earlier: R^2^ = 0.86 vs R^2^ = 0.88. This is likely because the majority of the mutations are missense mutations (88%) so when we build a pan mutation model it is mostly built for missense mutations. For nonsense mutations R^2^ for the pan-mutation was 0.34 while R^2^ for the nonsense-specific model was higher - R^2^ = 0.46. The type-specific model was also more accurate for frameshift mutations R^2^ = 0.22 versus R^2^ = 0.16. Therefore, the pan-mutation model works well for missense mutations, but for nonsense and frameshift mutations type-specific models perform better.

### Additional gene characteristics to improve the prediction accuracy of MutsigCV

MutsigCV is one of the most popular and efficient tool for identification of cancer genes from mutation data [27]. MutsigCV predicts the number of mutations in a gene based on the gene size and the number of silent mutations detected in a given set of tumor samples. Three other characteristics, “expr” – gene expression, “hic” – open chromatin and “reptime” – relative replication time are used as co variates. We tested if the inclusion of additional gene characteristics could improve prediction accuracy of MutsigCV. We used MutsigCV to identify cancer genes for analyses three different TCGA datasets: LUAD (Lung adenocarcinoma), LUSC (lung squamous cell carcinoma) and SKCM (skin cutaneous melanoma) with similar results. Here we show the results generated by an analysis of LUAD data as an example. MutsigCV identified ten lung adenocarcinoma associated genes: *KRAS, TP53, STK11, KEAP1, SMARCA4, EGFR, RBM10, C3orf27, ZNF831,* and *OR5M11*. Stepwise multivariate mutation-specific regression models identified a partially overlapping set of 21 cancer-associated genes: *EGFR, TP53, KRAS, SI, STK11, FLG, PTPRD, COL11A1, LRP1B, FBN2, NEIL3, CSMD3, SPTA1, CDH10, PCLO, MYH1, USH2A, SPHKAP, ZNF804A, XIRP2,* and *ZNF831*.

We tested if inclusion of additional gene characteristics identified in our study improves the prediction accuracy of MutsigCV. The inclusion of the nucleotide composition, the nucleotide diversity, gene expression, and the replication time only slightly improved R^2^ compared to the set of predictors used by MutsigCV: 0.60 versus 0.58. Hovewer, adding the number of silent mutations reported by genome wide screens in COSMIC led to substantial improvement in prediction efficacy: 0.66 vs 0.58. Similar results were obtained for LUSC and SKCM data. Therefore, incorporating the number of silent mutations reported by genome wide screens across different cancer types can significantly improve prediction accuracy of MutsigCV.

### Genes with a higher than expected number of mutations (positive outliers)

We identified 111 positive outliers - genes with a significant excess of missense, nonsense, or frameshift mutations, after the adjustment for multiple testing (Additional file 10). *TP53* and *PTEN* have a higher than expected number of all three types of mutations. Five genes, *ATM, LRP1B, CSMD3, FBXW*, and *SMAD4* have an excess of missense and nonsense mutations. Three genes, *COL11A1*, *SLC25A5*, and *PCLO* show a significant excess of frameshift and missense mutations. Twelve genes: *APC, AXIN1, TET2, ASXL1, ARID2, RB1, NF1, VHL, PBRM1, KMT2D, KMT2C*, and *ARID1A,* show an excess of frameshift and nonsense mutations.

### Z-scores for known cancer-associated genes

**Fig. 4:**
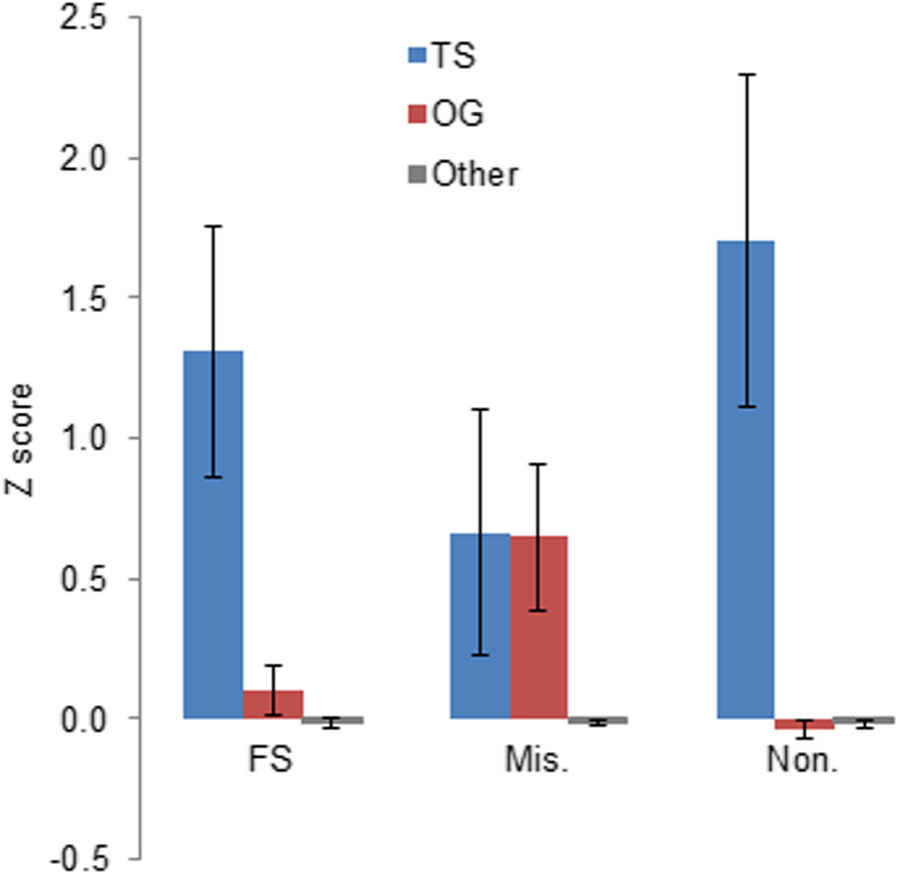
Z-scores for known tumor suppressor genes (TS), oncogenes (OG) and the genes that are not reported by UniprotKB as TS or OG – other genes. Z-scores for FS, missense (Mis.) and nonsense (Non.) mutations are shown separately. Vertical bars indicate the standard error of mean

We computed Z-scores for known tumor suppressor genes (TS) and oncogenes (OGs) and compared them with Z-scores for other genes in the human genome. TS and OGs were defined by UniprotKB database [28, 29]. There are 233 OGs and 176 TSs. Genes that are not reported as TSs or OGs (other genes) were used as a reference group. The mean Z-score for known TSs was significantly higher for FS, missense, and nonsense mutations compared to Z-scores for all other genes. For known OGs the mean Z-score was higher for missense mutations only (Fig. 4). A higher Z-score for missense mutations is expected because typically activating missense mutations in oncogenes drive tumorigenesis. [30, 31].

### Major findings

We found that gene characteristics can explain considerable proportion of inter genic variation in the number of somatic mutations: 88% for missense, 40% for nonsense, and 23% for frameshift mutations. Many genes with a higher-than-expected number of mutations (positive outliers) were also identified. Over hundred positive outliers were not previously reported by the COSMIC cancer consensus database and therefore can be considered as novel candidate cancer genes.

## Discussion

A goal of this study was to identify gene characteristics associated with the number of somatic mutations in tumor samples. Since gene characteristics we used as predictors are inter-correlated, we applied stepwise best subset regression model. Regression models explain 88% of variation in the number of missense, 40% nonsense, and 23% of frameshift mutations. If we assume that the unexplained variation in the number of mutations is due to an involvement of the gene in cancer development, the results show that FS most frequently associated with tumorigenesis followed by nonsense and missense mutations.

Each gene in the human genome acquires mutations on background level based on intrinsic mutability of the gene which depends on gene characteristics. Cancer associated genes are expected to have extra mutations due to selection of clones with driver mutations. In our analysis positive outliers (genes with a higher-than-expected number of mutations) were considered as candidate cancer associated genes. The majority of outliers are known cancer-associated genes. We also identified a number of novel putative cancer-associated genes. We considered a gene as a novel cancer-associated gene when the following three criteria were satisfied: the gene is not listed among (1) COSMIC cancer census genes; (2) Mayo Clinic 50 gene cancer panel [32] or (3) Foundation Medicine 315 gene panel. We have identified 18 novel cancer-associated genes with an excess of missense mutations: *MUC4, CSMD3, FLG, USH2A, DNAH8, FAT4, MUC17, MUC16, SYNE1, COL11A1, RP1, SI, SACS, SLC25A5, DMD, DST, XIRP2,* and *PKHD1L1.* We also identified 67 genes with an excess of FS and/or nonsense mutations: *ACVR2A, SOX9, RPL22, CDCP2, CRIPAK, FAT1, BAX, BCL9L, SON, TTK, ZFP36L2, RBMX, XYLT2, USP35, WBP1, BMPR2, ZDBF2, MBD6, TCF7L2, PABPC3, ESRP1, ZC3H18, TDG, SLC23A2, JPH4, UBR5, PDS5B, IL32, BCL9, SYCP1, PRRT2, ROBO2, TEAD2, ZNF626, CASP8, RBM10, WNT16, PTCHD3, CD3G, RTKN2, PLEKHA6, AKAP7, DDX27, SEC63, ADNP, NKTR, NDUFC2, MANEA, SYNJ2, TMEM60, ARV1, LARP4B, PHACTR4, TBX3, HNRNPL, PRRG1, MCPH1, CEP290, MAP7D1, CCDC73, GPATCH4, TGIF1, FAM111B, CLOCK, SCLT1, HOXB3,* and *SRRT*. A larger number of novel cancer-associated genes identified through the analyses of FS and nonsense mutilations compared to the analysis of missense mutations can be due to the fact that a large proportion of variation in number of mutation is due to gene involvement in cancer development.

For some genes in the human genome, the total number of missense mutations does not differ significantly from the expected number, hovewer, those mutations are clustered. For example, the observed number of missense mutations in *AKT1* oncogene is 113. This does not differ significantly from the expected number of the mutations (70), Z_(M)_ = 0.86. However, the majority (86 out of 113) of the mutation counts are p.E17K mutation. If we exclude p.E17K, in the reminder of the *AKT1* gene the observed number of mutations is lower than expected: 27 observed versus 70 expected. The lower number of mutations in the rest of the gene may be due to the fact that most of the coding region (85%) is occupied by functional domains. Missense mutations in functional domains may be loss-of-function mutations and as a result are negatively selected in tumors. Because our modeling does not take into account the distribution of mutations within the coding region, it may miss cancer genes with a clustering of functional mutations but a similar number of observed and expected mutations.

Interestingly, many novel cancer-associated genes identified by the excess of missense mutations are large genes with repetitive functional domains: *LRP1B, CSMD3, FLG, USH2A* and others. In these genes functional mutations tend to be uniformly distributed across repetitive functional domains. For example, one of the frequent mutations in *CSMD3* gene is G > A substitution. It leads to arginine (R) to glutamine (Q) substitution. The mutation is reported at position 11 of the repetitive sushi domain: sushi domain #5 (2 mutations), sushi domain #7 (4 mutations), sushi domain #9 (7 mutations), and sushi domain #13 (6 mutations). Taking into account that 92% of mutations in the gene are singletons, the observed pattern is likely to reflect the existence of multiple peaks distributed across repetitive functional domains.

We found that a small number of gene characteristics predict a large part of variation in the number of mutations per gene. “Number of silent mutations in the gene” alone explains 84.3% of variation in the number of missense mutations per gene. Adding “Percentage of “C”” and “Nucleotide diversity” improves prediction accuracy to 85.7 and 85.8% correspondingly. Adding last four predictors listed in Table 8 increases R^2^ from 85.7 to 88.1%. Therefore, the first three predictors explain most of the variation in the number of missense mutations per gene.

For nonsense mutations, the number of potential sites for nonsense substitutions alone explains 34.7% of variation. Adding the number of silent mutations in the gene as a predictor increases R^2^ to 37.4%. Adding the gene size as a predictor further increases R^2^ to 39.4%. Including all significant predictors listed in Table 8 makes R^2^ equal to 39.6%.

For frameshift mutations, the gene size alone explains 21.6% of variation. Adding 8 other significant predictors listed in Table 10 leads to only an incremental increase in R^2^ to 22.8%.

We found that the number of silent mutations reported by COSMIC genome wide screens across all cancer types is the most significant predictor of missense mutations. It also contributed significantly to the prediction of nonsense as well as frameshift mutations. The number of silent mutations is the most important predictor of the number of somatic mutations in the gene because it is an integrative indicator of the background mutability of the gene.

The strongest predictor of nonsense mutations was the number of potential sites for that type of substitutions. It explains 34.7% of total variation. Only 21 out of possible 64 codons are capable of producing nonsense mutations by SNSs. The number of potential sites for nonsense mutation varies an order of magnitude across genes, from 0.03 per nucleotide for *MUC21* to 0.29 for *KRTAP20–1*. The ability of the gene to generate nonsense mutations depends on codon composition.

We also found that the total number of silent mutations per gene reported by genome screens in COSMIC across different cancers improves the predicting accuracy of MutsigCV. MutsigCV uses the number of silent mutations in analyzed set of tumor samples as a predictor. The number of silent mutations in a single sample tends to have a large variation because the typical sample size is small. Also different cancer types tend to have different mutation spectra (mutation signature). [33] An underestimation of the number of silent mutations in a sample can lead to false positives by MutsigCV but not by our analysis. In our analysis of LUAD data, MutsigCV identified “Chromosome 3 Open Reading Frame 27” (*C3orf27)* as statistically significant with adjusted *P*-value of 0.02. The *C3orf27* is an unexpected candidate: it is a small gene with no evidence reported to date that it is cancer related. There are no reported silent mutations in the gene in LUAD sample which implies that the overall mutability of the gene is low suggesting non-silent mutations in the gene are cancer related. Based on COSMIC data, *C3orf27* has a ratio of silent to non-silent mutations of 0.21, which does not differ significantly from the average ratio of 0.34. In our regression model *C3orf27* was not significant. Therefore, the total number of silent mutations per gene generated by whole genome (exome) mutational screens across different cancer types is a key predictor of somatic mutations and needs to be included in cancer gene prediction models including MutsigCV to increase the specificity of the results.

We found that top predictors for missense, nonsense and FS mutations are different. As a result, the mutation-type specific prediction models work better for identification of cancer-associated genes compared to the pan-mutation model. Though the pan-mutation model performs acceptably in predicting the number of missense mutations, its prediction accuracy for nonsense and frameshift mutations is poor compared to the mutation-specific models.

## Conclusions

We analyzed a number of gene characteristics associated with missense, nonsense, and frameshift mutations. We applied stepwise best subset multivariate model to predict missense, nonsense, and FS mutations using gene characteristics, and by comparison of the observed and expected number of mutations identified novel cancer-associated genes. We showed that including the total number of silent mutations per gene identified by whole genome/exome screens across different cancer types led to a substantial improvement in the prediction efficacy, indicating that this variable needs to be included in existing prediction algorithms, e.g. MutsigCV. We also generated a list of novel candidate cancer-associated genes that may warrant further analysis.

## Additional files

Additional file 1: The relationship between the number of potential sites for a given type of mutations and the number of the mutations of the same type. As expected, there is a strong positive association between the number of potential sites and the number of reported mutations similar to what was observed for the gene size. (DOCX 1370 kb)

Additional file 2: Olfactory genes and densities of missense mutations. First row shows the proportion of olfactory genes in a sliding window of 100 genes, when it moves from lowest to highest nucleotide content. Second row shows the distribution of olfactory (red) and other (blue) genes across mutation densities bins. Olfactory genes have a higher density of missense but not nonsense mutations. Third row shows the effect of excluding of olfactory genes on the relationship between percentage of “T” in the gene and density of missense mutations. (DOCX 2413 kb)

Additional file 3: The relationship between the proportion of CpG sites and the mutation densities. Proportion of CpGs was computed as the ratio of the number of CpGs in the gene to the gene size in nucleotides. (DOCX 143 kb)

Additional file 4: The relationship between conservation index and the mutation density. (DOCX 1044 kb)

Additional file 5: The relationship between nucleotide diversity of the gene sequences and the densities of somatic mutations. (DOCX 146 kb)

Additional file 6: The relationship between chromatin accessibility and the mutation densities for missense, nonsense and frameshift mutations. (DOCX 130 kb)

Additional file 7: The relationship between the observed and expected number of missense mutations. Each dot represents a gene. (DOCX 566 kb)

Additional file 8: The relationship between the observed and expected number of nonsense mutations. Each dot represents a gene. (DOCX 654 kb)

Additional file 9: The relationship between the observed and expected number of frameshift mutations. Each dot represents a gene. (DOCX 654 kb)

Additional file 10: Genes with a higher than expected number of frameshift, missense, or nonsense mutations. Genes sorted on the maximum Z value. (DOCX 51 kb)

## Abbreviations

CCLE: Cancer Cell Line Encyclopedia
COSMIC: Catalog of Somatic Mutations in Cancer
FS: Frameshift mutations
LUAD: Lung adenocarcinoma
LUSC: Lung squamous cell carcinoma
ND: Nucleotide diversity
OG: Oncogene
SKCM: Skin cutaneous melanoma
SNS: Single nucleotide substitution
TS: Tumor suppressors
