# Supplementary material for: Gene characteristics predicting missense, nonsense and frameshift mutations in tumor samples
Source: BMC Bioinformatics. 2018 Nov 19;19:430. doi: 10.1186/s12859-018-2455-0 (PMC6245819; doi:10.1186/s12859-018-2455-0)
Supplement: Supplementary file 1 — The relationship between the number of potential sites for a given type of mutations and the number of the mutations of the same type. As expected, there is a strong positive association between the number of potential sites and the number of reported mutations similar to what was observed for the gene size. (DOCX 1370 kb) [file 12859_2018_2455_MOESM1_ESM.docx]

**
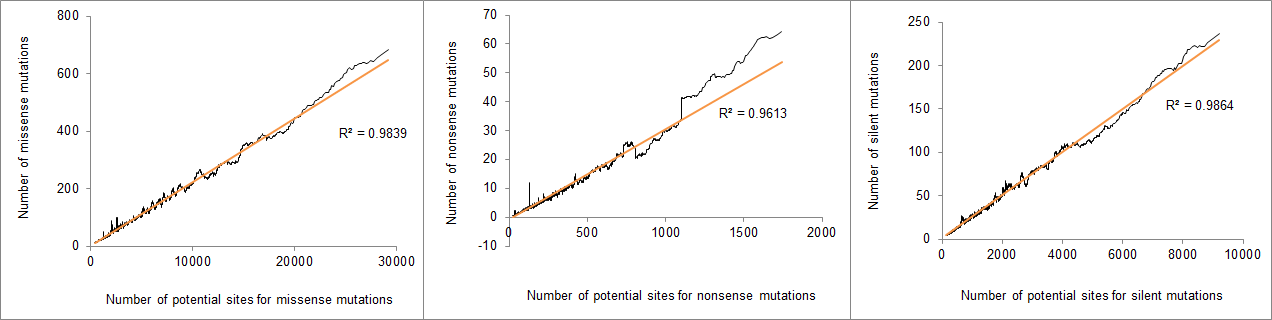
**

**Additional file 1:** The relationship between the number of potential sites for a given type of mutations and the number of the mutations of the same type. As expected, there is a strong positive association between the number of potential sites and the number of reported mutations similar to what was observed for the gene size.
