# Supplementary material for: Gene characteristics predicting missense, nonsense and frameshift mutations in tumor samples
Source: BMC Bioinformatics. 2018 Nov 19;19:430. doi: 10.1186/s12859-018-2455-0 (PMC6245819; doi:10.1186/s12859-018-2455-0)
Supplement: Supplementary file 2 — Olfactory genes and densities of missense mutations. First row shows the proportion of olfactory genes in a sliding window of 100 genes, when it moves from lowest to highest nucleotide content. Second row shows the distribution of olfactory (red) and other (blue) genes across mutation densities bins. Olfactory genes have a higher density of missense but not nonsense mutations. Third row shows the effect of excluding of olfactory genes on the relationship between percentage of “T” in the gene and density of missense mutations. (DOCX 2413 kb) [file 12859_2018_2455_MOESM2_ESM.docx]

**
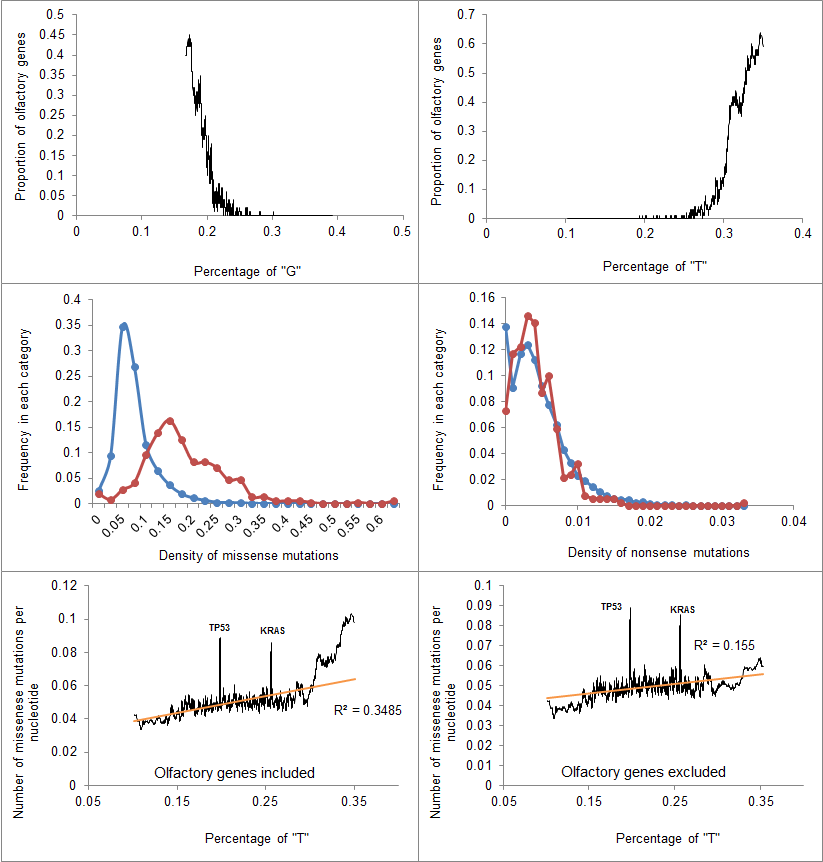
**

**Additional file 2:** Olfactory genes and densities of missense mutations. **First row** shows the proportion of olfactory genes in a sliding window of 100 genes, when it moves from lowest to highest nucleotide content. **Second row** shows the distribution of olfactory (red) and other (blue) genes across mutation densities bins. Olfactory genes have a higher density of missense but not nonsense mutations. **Third row** shows the effect of excluding of olfactory genes on the relationship between percentage of “T” in the gene and density of missense mutations.

We observed up-rising tails (left for percentage of “G”, and right for percentage of “T”) for the curves describing the relationship between the densities of missense mutations and nucleotide composition. The tails are enriched by olfactory genes. Figure S2 (upper panel) shows the proportion of olfactory genes within a sliding window of 100 genes when it moves across the genes ranked by nucleotide content. The percentage of “C” in olfactory genes is similar to that of other genes: 0.27±0.01 vs 0.26±0.01, while the percentage of “A” in olfactory genes is slightly lower: 0.22±0.01 vs 0.25±0.01. Olfactory genes have a higher density of missense mutations compared to other genes: 0.11±0.01 vs 0.04±0.01. Densities of nonsense mutations are similar in olfactory and other genes (middle panel). Based on the unique nucleotide composition and mutation densities we excluded olfactory genes from the model building which led to disappearance of the up-rising tails (lower panel).
