# Supplementary material for: Gene characteristics predicting missense, nonsense and frameshift mutations in tumor samples
Source: BMC Bioinformatics. 2018 Nov 19;19:430. doi: 10.1186/s12859-018-2455-0 (PMC6245819; doi:10.1186/s12859-018-2455-0)
Supplement: Supplementary file 3 — The relationship between the proportion of CpG sites and the mutation densities. Proportion of CpGs was computed as the ratio of the number of CpGs in the gene to the gene size in nucleotides. (DOCX 143 kb) [file 12859_2018_2455_MOESM3_ESM.docx]

**
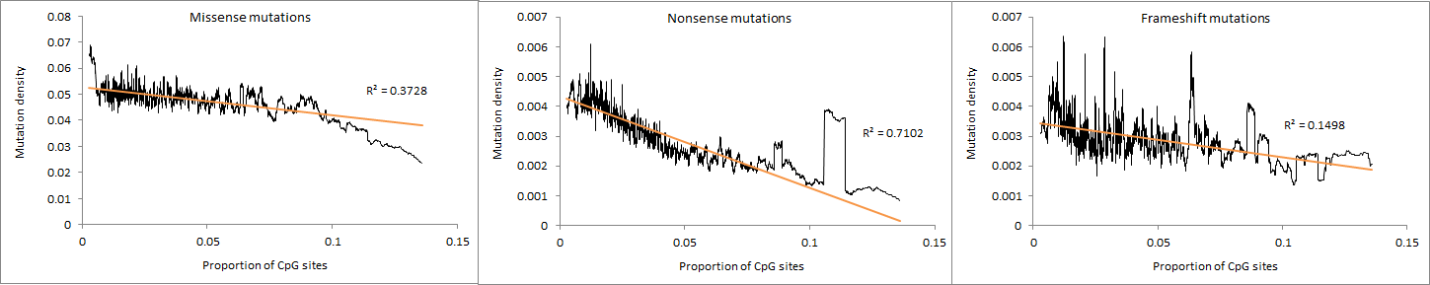
**

**Additional file 3:** The relationship between the proportion of CpG sites and the mutation densities. Proportion of CpGs was computed as the ratio of the number of CpGs in the gene to the gene size in nucleotides.

For missense and nonsense mutations we have observed a negative association between the percentage of CpGs and mutation density.
