# Supplementary material for: Gene characteristics predicting missense, nonsense and frameshift mutations in tumor samples
Source: BMC Bioinformatics. 2018 Nov 19;19:430. doi: 10.1186/s12859-018-2455-0 (PMC6245819; doi:10.1186/s12859-018-2455-0)
Supplement: Supplementary file 4 — The relationship between conservation index and the mutation density. (DOCX 1044 kb) [file 12859_2018_2455_MOESM4_ESM.docx]

**
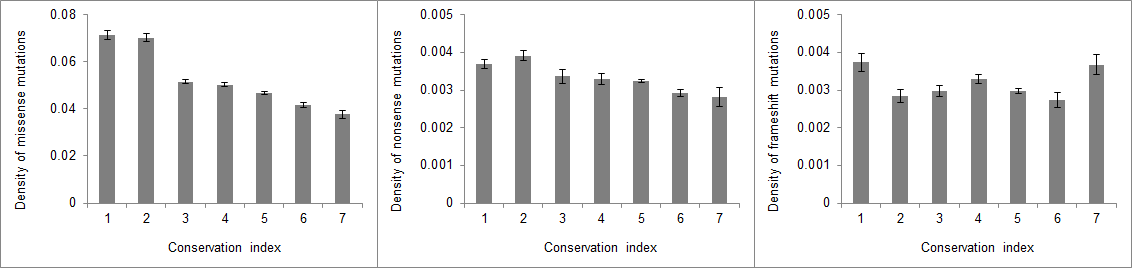
**

**Additional file 4:** The relationship between conservation index and the mutation density.

For missense and nonsense mutations there was a negative association between the level of evolutionary conservation and the mutation density. For frameshift mutations we did not observe a significant association between evolutionary conservation of the gene and the mutation density.
