# Supplementary material for: Gene characteristics predicting missense, nonsense and frameshift mutations in tumor samples
Source: BMC Bioinformatics. 2018 Nov 19;19:430. doi: 10.1186/s12859-018-2455-0 (PMC6245819; doi:10.1186/s12859-018-2455-0)
Supplement: Supplementary file 5 — The relationship between nucleotide diversity of the gene sequences and the densities of somatic mutations. (DOCX 146 kb) [file 12859_2018_2455_MOESM5_ESM.docx]

**
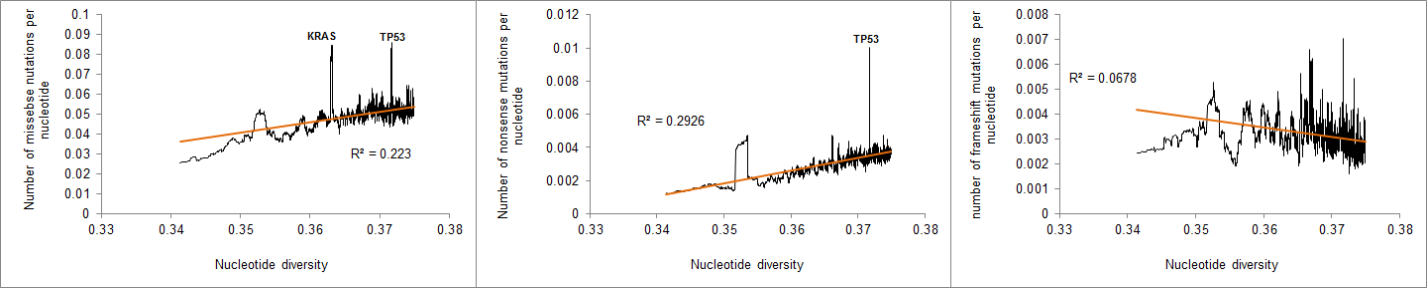
**

**Additional file 5:** The relationship between nucleotide diversity of the gene sequences and the densities of somatic mutations.

For missense, and nonsense mutations the relationships were positive and significant. For frameshift mutations the correlations was not significant.
