# Supplementary material for: Gene characteristics predicting missense, nonsense and frameshift mutations in tumor samples
Source: BMC Bioinformatics. 2018 Nov 19;19:430. doi: 10.1186/s12859-018-2455-0 (PMC6245819; doi:10.1186/s12859-018-2455-0)
Supplement: Supplementary file 6 — The relationship between chromatin accessibility and the mutation densities for missense, nonsense and frameshift mutations. (DOCX 130 kb) [file 12859_2018_2455_MOESM6_ESM.docx]

**
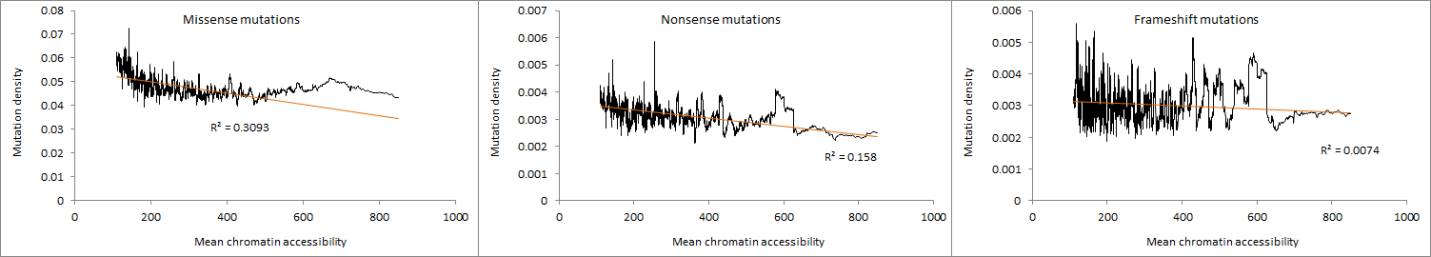
**

**Additional file 6:** The relationship between chromatin accessibility and the mutation densities for missense, nonsense and frameshift mutations.

A significant negative association between chromatin accessibility and the density of missense and nonsense mutations in the gene has been observed. The density of frameshift mutations showed no association with chromatin accessibility.
