# Supplementary material for: Gene characteristics predicting missense, nonsense and frameshift mutations in tumor samples
Source: BMC Bioinformatics. 2018 Nov 19;19:430. doi: 10.1186/s12859-018-2455-0 (PMC6245819; doi:10.1186/s12859-018-2455-0)
Supplement: Supplementary file 8 — The relationship between the observed and expected number of nonsense mutations. Each dot represents a gene. (DOCX 654 kb) [file 12859_2018_2455_MOESM8_ESM.docx]

**
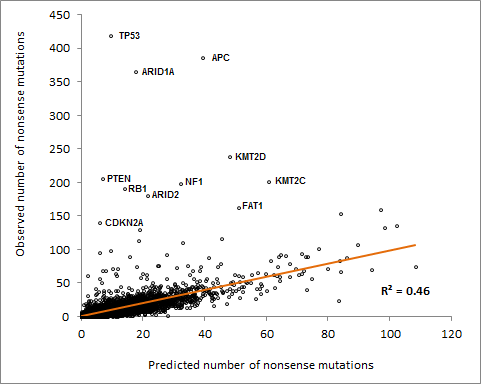
**

**Additional file 8:** The relationship between the observed and expected number of nonsense mutations. Each dot represents a gene.

For a number of genes the observed number of nonsense mutations was much higher compared to the number expected based on the gene characteristics.
