# Supplementary material for: Gene characteristics predicting missense, nonsense and frameshift mutations in tumor samples
Source: BMC Bioinformatics. 2018 Nov 19;19:430. doi: 10.1186/s12859-018-2455-0 (PMC6245819; doi:10.1186/s12859-018-2455-0)
Supplement: Supplementary file 10 — Genes with a higher than expected number of frameshift, missense, or nonsense mutations. Genes sorted on the maximum Z value. (DOCX 51 kb) [file 12859_2018_2455_MOESM10_ESM.docx]

**Additional file 10.** Genes with a higher than expected number of frameshift, missense, or nonsense mutations. Genes sorted on the maximum Z value.

Gene – official gene name. Gene ID – Entrez gene ID, Size – the size of the coding region in nucleotides, O(FS) – the observed number of frameshift mutations, E(FS) – the expected number of frameshift mutations, Z(FS) – Z score for residuals of frameshift mutations, O(M) – the observed number of missense mutations, E(M) – the expected number of missense mutations, Z(M) – Z score for residuals of missense mutations, O(N) – the observed number of nonsense mutations, E(N) – the expected number of nonsense mutations, Z(N) – Z score for residuals of nonsense mutations.

| Gene | Gene ID | Size | O(FS) | E(FS) | Z(FS) | O(M) | E(M) | Z(M) | O(N) | E(N) | Z(N) |
| --- | --- | --- | --- | --- | --- | --- | --- | --- | --- | --- | --- |
| TP53 | 7157 | 1182 | 335 | 2.80 | **48.74** | 4267 | 191.26 | **80.75** | 919 | 9.46 | **80.40** |
| APC | 324 | 8532 | 282 | 16.90 | **38.90** | 405 | 282.01 | 2.44 | 727 | 39.42 | **60.78** |
| KRAS | 3845 | 570 | 0 | 2.43 | -0.36 | 1856 | 77.57 | **35.23** | 2 | 2.62 | -0.05 |
| PIK3CA | 5290 | 3207 | 4 | 6.82 | -0.41 | 1842 | 132.41 | **33.87** | 11 | 15.35 | -0.38 |
| ARID1A | 8289 | 6858 | 186 | 12.94 | **25.39** | 346 | 195.90 | 2.97 | 366 | 17.54 | **30.80** |
| GATA3 | 2625 | 1335 | 133 | 4.80 | **18.81** | 108 | 131.63 | -0.47 | 5 | 6.22 | -0.11 |
| ACVR2A | 92 | 1542 | 128 | 3.05 | **18.33** | 117 | 69.54 | 0.94 | 36 | 7.06 | 2.56 |
| BRAF | 673 | 2301 | 5 | 4.27 | 0.11 | 1041 | 116.16 | **18.32** | 11 | 10.36 | 0.06 |
| PTEN | 5728 | 1212 | 125 | 4.37 | **17.70** | 465 | 93.91 | **7.35** | 206 | 6.73 | **17.61** |
| KMT2D | 8085 | 16614 | 109 | 29.74 | **11.63** | 708 | 601.56 | 2.11 | 238 | 48.21 | **16.78** |
| RB1 | 5925 | 2787 | 53 | 6.91 | **6.76** | 153 | 130.58 | 0.44 | 191 | 13.87 | **15.66** |
| RNF43 | 54894 | 2352 | 108 | 4.80 | **15.14** | 164 | 92.47 | 1.42 | 50 | 6.52 | 3.84 |
| NF1 | 4763 | 8520 | 66 | 15.69 | **7.38** | 409 | 317.04 | 1.82 | 199 | 32.15 | **14.75** |
| ARID2 | 196528 | 5508 | 54 | 10.83 | **6.33** | 297 | 215.92 | 1.61 | 180 | 21.48 | **14.01** |
| CTNNB1 | 1499 | 2346 | 1 | 4.10 | -0.45 | 808 | 120.08 | **13.63** | 14 | 7.71 | 0.56 |
| KMT2C | 58508 | 14736 | 108 | 26.50 | **11.96** | 699 | 522.05 | 3.51 | 202 | 60.58 | **12.50** |
| SOX9 | 6662 | 1530 | 89 | 5.28 | **12.28** | 88 | 50.13 | 0.75 | 22 | 4.07 | 1.58 |
| NPM1 | 4869 | 885 | 86 | 3.58 | **12.09** | 25 | 57.97 | -0.65 | 1 | 3.48 | -0.22 |
| CDKN2A | 1029 | 504 | 29 | 0.70 | 4.15 | 182 | 179.60 | 0.05 | 140 | 5.85 | **11.86** |
| RPL22 | 6146 | 387 | 81 | 2.05 | **11.58** | 29 | 22.82 | 0.12 | 5 | 0.87 | 0.36 |
| LRP1B | 53353 | 13800 | 23 | 24.08 | -0.16 | 1794 | 1220.04 | **11.37** | 159 | 97.23 | **5.46** |
| PBRM1 | 55193 | 4749 | 85 | 9.28 | **11.11** | 196 | 127.08 | 1.37 | 129 | 18.97 | **9.73** |
| CDCP2 | 200008 | 1350 | 75 | 3.04 | **10.56** | 58 | 84.65 | -0.53 | 2 | 4.25 | -0.20 |
| MUC4 | 4585 | 16239 | 8 | 32.02 | -3.52 | 1299 | 785.41 | **10.17** | 38 | 23.48 | 1.28 |
| CRIPAK | 285464 | 1341 | 72 | 3.00 | **10.12** | 104 | 101.19 | 0.06 | 7 | 5.33 | 0.15 |
| FAT1 | 2195 | 13767 | 41 | 24.71 | 2.39 | 664 | 642.79 | 0.42 | 163 | 50.97 | **9.90** |
| CDH1 | 999 | 2649 | 72 | 4.87 | **9.85** | 185 | 99.21 | 1.70 | 56 | 5.76 | 4.44 |
| NRAS | 4893 | 570 | 0 | 1.81 | -0.27 | 524 | 45.77 | **9.47** | 4 | 1.82 | 0.19 |
| IDH1 | 3417 | 1245 | 3 | 2.86 | 0.02 | 527 | 59.65 | **9.26** | 6 | 5.15 | 0.07 |
| BAX | 581 | 657 | 60 | 1.15 | **8.64** | 25 | 6.54 | 0.37 | 6 | 0.09 | 0.52 |
| CSMD3 | 114788 | 11124 | 35 | 19.55 | 2.27 | 1686 | 1251.88 | **8.60** | 154 | 84.08 | **6.18** |
| PCLO | 27445 | 15429 | 61 | 28.79 | **4.73** | 1694 | 1267.11 | **8.46** | 133 | 98.28 | 3.07 |
| KDM6A | 7403 | 4206 | 38 | 8.08 | 4.39 | 191 | 145.26 | 0.91 | 113 | 18.52 | **8.35** |
| BCL9L | 283149 | 4500 | 66 | 9.74 | **8.25** | 203 | 178.87 | 0.48 | 8 | 6.15 | 0.16 |
| SON | 6651 | 7281 | 69 | 13.20 | **8.19** | 277 | 287.28 | -0.20 | 2 | 28.04 | -2.30 |
| TTK | 7272 | 2574 | 63 | 7.41 | **8.16** | 118 | 126.57 | -0.17 | 8 | 14.75 | -0.60 |
| ZFP36L2 | 678 | 1485 | 59 | 4.63 | **7.98** | 78 | 24.60 | 1.06 | 7 | 0.06 | 0.61 |
| SMAD4 | 4089 | 1659 | 32 | 2.85 | 4.28 | 330 | 70.21 | **5.15** | 96 | 6.02 | **7.95** |
| FBXW7 | 55294 | 2124 | 25 | 4.44 | 3.02 | 379 | 91.66 | **5.69** | 99 | 9.37 | **7.92** |
| RBMX | 27316 | 1176 | 56 | 2.17 | **7.90** | 126 | 62.48 | 1.26 | 4 | 6.00 | -0.18 |
| VHL | 7428 | 642 | 53 | 0.06 | **7.77** | 210 | 39.14 | 3.39 | 61 | 1.90 | **5.22** |
| XYLT2 | 64132 | 2598 | 57 | 4.67 | **7.68** | 75 | 71.39 | 0.07 | 1 | 4.35 | -0.30 |
| USP35 | 57558 | 3057 | 56 | 5.00 | **7.48** | 101 | 128.37 | -0.54 | 3 | 8.04 | -0.45 |
| WBP1 | 23559 | 810 | 53 | 2.14 | **7.46** | 31 | 12.88 | 0.36 | 2 | 1.26 | 0.07 |
| FLG | 2312 | 12186 | 18 | 22.00 | -0.59 | 1349 | 982.10 | **7.27** | 83 | 83.65 | -0.06 |
| BMPR2 | 659 | 3117 | 54 | 6.33 | **6.99** | 126 | 89.70 | 0.72 | 29 | 9.49 | 1.72 |
| USH2A | 7399 | 15609 | 20 | 27.40 | -1.09 | 1478 | 1125.87 | **6.98** | 107 | 89.52 | 1.54 |
| ZDBF2 | 57683 | 7065 | 61 | 13.56 | **6.96** | 446 | 377.83 | 1.35 | 44 | 36.05 | 0.70 |
| MBD6 | 114785 | 3012 | 55 | 7.65 | **6.95** | 120 | 99.51 | 0.41 | 16 | 2.12 | 1.23 |
| TCF7L2 | 6934 | 1809 | 52 | 4.65 | **6.95** | 124 | 115.79 | 0.16 | 27 | 8.67 | 1.62 |
| SETD2 | 29072 | 7695 | 37 | 14.99 | 3.23 | 344 | 171.01 | 3.43 | 111 | 32.78 | **6.91** |
| PABPC3 | 5042 | 1896 | 49 | 3.69 | **6.65** | 223 | 119.07 | 2.06 | 8 | 6.55 | 0.13 |
| ASXL1 | 171023 | 4626 | 45 | 8.26 | **5.39** | 187 | 228.87 | -0.83 | 90 | 15.54 | **6.58** |
| TGFBR2 | 7048 | 1779 | 47 | 3.16 | **6.43** | 167 | 82.60 | 1.67 | 26 | 5.98 | 1.77 |
| ESRP1 | 54845 | 2046 | 47 | 3.54 | **6.38** | 117 | 101.25 | 0.31 | 16 | 7.59 | 0.74 |
| DNAH8 | 1769 | 14124 | 23 | 25.18 | -0.32 | 1002 | 682.29 | **6.33** | 90 | 72.82 | 1.52 |
| ATM | 472 | 9171 | 35 | 17.54 | 2.56 | 531 | 245.50 | **5.66** | 117 | 45.50 | **6.32** |
| ZC3H18 | 124245 | 2934 | 48 | 5.42 | **6.25** | 106 | 110.88 | -0.10 | 15 | 9.96 | 0.45 |
| TDG | 6996 | 1233 | 46 | 4.00 | **6.16** | 56 | 55.78 | 0.00 | 3 | 5.48 | -0.22 |
| SLC23A2 | 9962 | 1953 | 45 | 3.00 | **6.16** | 85 | 77.14 | 0.16 | 2 | 5.11 | -0.27 |
| FAT4 | 79633 | 14946 | 22 | 26.16 | -0.61 | 1417 | 1108.57 | **6.11** | 91 | 66.25 | 2.19 |
| BCORL1 | 63035 | 5136 | 51 | 9.42 | **6.10** | 251 | 257.49 | -0.13 | 24 | 14.53 | 0.84 |
| MUC17 | 140453 | 13482 | 6 | 25.37 | -2.84 | 1201 | 893.10 | **6.10** | 42 | 34.81 | 0.64 |
| JPH4 | 84502 | 1887 | 45 | 3.53 | **6.09** | 83 | 72.33 | 0.21 | 3 | 3.56 | -0.05 |
| MUC16 | 94025 | 43524 | 37 | 78.06 | -6.02 | 4162 | 3855.17 | **6.08** | 184 | 189.72 | -0.51 |
| UBR5 | 51366 | 8400 | 56 | 15.08 | **6.00** | 307 | 262.44 | 0.88 | 30 | 30.70 | -0.06 |
| PDS5B | 23047 | 4344 | 50 | 9.37 | **5.96** | 158 | 157.20 | 0.02 | 22 | 20.14 | 0.16 |
| IL32 | 9235 | 705 | 41 | 0.52 | **5.94** | 22 | 28.00 | -0.12 | 1 | 2.28 | -0.11 |
| BCL9 | 607 | 4281 | 49 | 8.67 | **5.92** | 202 | 159.09 | 0.85 | 10 | 6.22 | 0.33 |
| SF3B1 | 23451 | 3915 | 2 | 7.78 | -0.85 | 413 | 114.80 | **5.91** | 10 | 14.90 | -0.43 |
| SYCP1 | 6847 | 2931 | 50 | 9.90 | **5.88** | 191 | 188.28 | 0.05 | 33 | 22.83 | 0.90 |
| PRRT2 | 112476 | 1185 | 42 | 3.01 | **5.72** | 36 | 29.44 | 0.13 | 1 | 1.11 | -0.01 |
| ROBO2 | 6092 | 4185 | 46 | 7.25 | **5.69** | 403 | 286.77 | 2.30 | 34 | 19.82 | 1.25 |
| CREBBP | 1387 | 7329 | 32 | 13.91 | 2.65 | 492 | 361.73 | 2.58 | 92 | 27.71 | **5.68** |
| SYNE1 | 23345 | 26394 | 20 | 46.59 | -3.90 | 1685 | 1400.96 | **5.63** | 158 | 152.46 | 0.49 |
| COL11A1 | 1301 | 5421 | 40 | 8.99 | **4.55** | 761 | 477.65 | **5.61** | 38 | 29.93 | 0.71 |
| TEAD2 | 8463 | 1356 | 40 | 1.90 | **5.59** | 67 | 70.98 | -0.08 | 3 | 4.35 | -0.12 |
| ZNF626 | 199777 | 1587 | 43 | 5.28 | **5.53** | 199 | 118.14 | 1.60 | 16 | 10.79 | 0.46 |
| CASP8 | 841 | 1617 | 11 | 3.46 | 1.11 | 147 | 66.27 | 1.60 | 69 | 6.94 | **5.49** |
| RBM10 | 8241 | 2988 | 10 | 5.65 | 0.64 | 118 | 99.78 | 0.36 | 72 | 10.21 | **5.46** |
| AXIN1 | 8312 | 2589 | 41 | 4.81 | **5.31** | 116 | 108.06 | 0.16 | 69 | 7.87 | **5.40** |
| WNT16 | 51384 | 1098 | 38 | 1.17 | **5.40** | 48 | 47.41 | 0.01 | 1 | 4.28 | -0.29 |
| PTCH1 | 5727 | 4344 | 44 | 7.20 | **5.40** | 232 | 254.98 | -0.46 | 29 | 16.68 | 1.09 |
| TET2 | 54790 | 6009 | 49 | 12.30 | **5.38** | 259 | 156.96 | 2.02 | 76 | 24.64 | **4.54** |
| PTCHD3 | 374308 | 2304 | 40 | 3.67 | **5.33** | 194 | 278.67 | -1.68 | 12 | 15.97 | -0.35 |
| RP1 | 6101 | 6471 | 22 | 12.58 | 1.38 | 850 | 581.89 | **5.31** | 51 | 42.66 | 0.74 |
| CD3G | 917 | 549 | 37 | 1.16 | **5.26** | 22 | 21.55 | 0.01 | 3 | 1.75 | 0.11 |
| AMER1 | 139285 | 3408 | 15 | 6.07 | 1.31 | 149 | 115.27 | 0.67 | 71 | 11.81 | **5.23** |
| RTKN2 | 219790 | 1830 | 40 | 4.35 | **5.23** | 76 | 96.21 | -0.40 | 17 | 8.50 | 0.75 |
| PLEKHA6 | 22874 | 3147 | 42 | 6.36 | **5.23** | 158 | 151.71 | 0.12 | 16 | 10.32 | 0.50 |
| AKAP7 | 9465 | 1047 | 39 | 3.51 | **5.21** | 41 | 64.89 | -0.47 | 5 | 4.64 | 0.03 |
| DDX27 | 55661 | 2391 | 40 | 4.62 | **5.19** | 96 | 107.62 | -0.23 | 11 | 8.75 | 0.20 |
| WT1 | 7490 | 1554 | 38 | 2.67 | **5.18** | 120 | 74.13 | 0.91 | 17 | 5.80 | 0.99 |
| SI | 6476 | 5484 | 11 | 10.49 | 0.07 | 696 | 434.48 | **5.18** | 69 | 36.01 | 2.92 |
| SEC63 | 11231 | 2283 | 41 | 5.79 | **5.17** | 84 | 74.05 | 0.20 | 9 | 11.95 | -0.26 |
| SACS | 26278 | 13740 | 50 | 25.65 | 3.57 | 714 | 454.07 | **5.15** | 74 | 63.68 | 0.91 |
| ADNP | 23394 | 3309 | 42 | 7.28 | **5.09** | 119 | 113.94 | 0.10 | 13 | 13.66 | -0.06 |
| NKTR | 4820 | 4389 | 44 | 9.56 | **5.05** | 135 | 106.18 | 0.57 | 9 | 21.66 | -1.12 |
| STAG2 | 10735 | 3807 | 25 | 7.96 | 2.50 | 174 | 174.76 | -0.02 | 75 | 18.00 | **5.04** |
| NDUFC2 | 4718 | 360 | 34 | -0.11 | **5.00** | 9 | 7.44 | 0.03 | 0 | 0.18 | -0.02 |
| SLC25A5 | 292 | 897 | 33 | 1.08 | **4.68** | 315 | 63.49 | **4.98** | 24 | 3.14 | 1.84 |
| MSH3 | 4437 | 3414 | 41 | 7.24 | **4.95** | 116 | 132.09 | -0.32 | 17 | 14.66 | 0.21 |
| MANEA | 79694 | 1389 | 38 | 4.41 | **4.93** | 82 | 67.05 | 0.30 | 7 | 6.69 | 0.03 |
| SYNJ2 | 8871 | 4491 | 41 | 7.54 | **4.91** | 164 | 190.38 | -0.52 | 6 | 13.48 | -0.66 |
| DMD | 1756 | 11058 | 18 | 20.26 | -0.33 | 806 | 558.49 | **4.90** | 92 | 64.46 | 2.43 |
| TMEM60 | 85025 | 402 | 35 | 1.60 | **4.90** | 9 | 41.00 | -0.63 | 0 | 1.81 | -0.16 |
| ARV1 | 64801 | 816 | 35 | 1.77 | **4.88** | 73 | 27.59 | 0.90 | 1 | 3.49 | -0.22 |
| DST | 667 | 16614 | 22 | 30.72 | -1.28 | 758 | 515.84 | **4.80** | 88 | 86.05 | 0.17 |
| LARP4B | 23185 | 2217 | 37 | 4.30 | **4.80** | 108 | 88.56 | 0.39 | 17 | 7.83 | 0.81 |
| XIRP2 | 129446 | 10650 | 23 | 20.54 | 0.36 | 1168 | 925.99 | **4.79** | 89 | 73.33 | 1.39 |
| PHACTR4 | 65979 | 2139 | 38 | 5.65 | **4.75** | 77 | 59.22 | 0.35 | 10 | 5.23 | 0.42 |
| TBX3 | 6926 | 2232 | 36 | 3.72 | **4.74** | 131 | 131.51 | -0.01 | 11 | 6.06 | 0.44 |
| HNRNPL | 3191 | 1770 | 35 | 2.73 | **4.74** | 73 | 70.80 | 0.04 | 5 | 4.23 | 0.07 |
| PRRG1 | 5638 | 657 | 34 | 2.02 | **4.69** | 47 | 61.01 | -0.28 | 1 | 3.47 | -0.22 |
| HNF1A | 6927 | 1917 | 37 | 5.06 | **4.69** | 127 | 121.40 | 0.11 | 6 | 5.42 | 0.05 |
| PIK3R1 | 5295 | 2175 | 27 | 5.30 | 3.18 | 169 | 86.30 | 1.64 | 64 | 11.03 | **4.68** |
| PKHD1L1 | 93035 | 12732 | 16 | 22.61 | -0.97 | 988 | 752.54 | **4.66** | 64 | 59.90 | 0.36 |
| MCPH1 | 79648 | 2508 | 37 | 5.23 | **4.66** | 89 | 114.61 | -0.51 | 5 | 12.12 | -0.63 |
| CEP290 | 80184 | 7440 | 49 | 17.24 | **4.66** | 231 | 223.77 | 0.14 | 38 | 47.18 | -0.81 |
| MAP7D1 | 55700 | 2526 | 38 | 6.25 | **4.66** | 85 | 67.94 | 0.34 | 6 | 5.69 | 0.03 |
| CCDC73 | 493860 | 3240 | 42 | 10.35 | **4.64** | 144 | 175.09 | -0.62 | 26 | 20.24 | 0.51 |
| GPATCH4 | 54865 | 1128 | 35 | 3.47 | **4.63** | 41 | 54.75 | -0.27 | 4 | 4.05 | 0.00 |
| TGIF1 | 7050 | 1206 | 34 | 2.63 | **4.60** | 55 | 19.03 | 0.71 | 6 | 1.01 | 0.44 |
| FAM111B | 374393 | 2205 | 37 | 5.70 | **4.59** | 97 | 113.72 | -0.33 | 5 | 10.79 | -0.51 |
| CLOCK | 9575 | 2541 | 37 | 5.95 | **4.56** | 81 | 68.22 | 0.25 | 6 | 10.29 | -0.38 |
| SCLT1 | 132320 | 2067 | 38 | 7.03 | **4.54** | 76 | 117.38 | -0.82 | 11 | 13.34 | -0.21 |
| HOXB3 | 3213 | 1296 | 35 | 4.05 | **4.54** | 66 | 111.62 | -0.90 | 7 | 5.38 | 0.14 |
| SRRT | 51593 | 2631 | 35 | 4.29 | **4.51** | 140 | 112.20 | 0.55 | 5 | 8.94 | -0.35 |
